# Detection of Hydrogen Peroxide Vapors Using Acidified Titanium(IV)-Based Test Strips

**DOI:** 10.3390/ma17235887

**Published:** 2024-12-01

**Authors:** Rayhan Hossain, Nicholas F. Materer

**Affiliations:** 1 Department of Natural Sciences, 107 Science Faculty Center, University of Michigan, Dearborn, MI 48128, USA; rhossain@umich.edu; 2 Department of Chemistry, 316 Physical Science, Oklahoma State University, Stillwater, OK 74078, USA

**Keywords:** titania, ionic liquid, hydrogen peroxide vapor, thin-film sensor, colorimetry

## Abstract

One method for the colorimetric detection of hydrogen peroxide vapor is based on a titanium–hydrogen peroxide complex. A color changing material based on a titania hydroxypropyl cellulose thin film was initially developed. However, as this material dries, the sensitivity of the material is significantly reduced. Thus, an alternative sensing material, based on titanium(IV) oxysulfate, an ionic liquid, and in some cases, triflouromethanesulfonic acid adsorbed onto low-cost silicon thin-layer chromatography (TLC) plates, was developed. TiO_2_ was heated with concentrated sulfuric acid in a controlled environment, usually at temperatures ranging from 100 °C to 250 °C. These sensors are disposable and single-use and are simple and inexpensive. When the resulting thin-film sensors are exposed to ppm levels of hydrogen peroxide vapor, they turn from a white reflective material to an intense yellow or orange. Ti(IV) oxysulfate combined with an acid catalyst and an ionic-liquid-based material provides an opportunity to enhance the sensor activity towards the peroxide vapor and decreases the detection limit. Kinetic measurements were made by the quantification of the intensity of the reflected light as a function of the exposure time from the sensor in a special cell using a low-cost web camera and a tungsten lamp. The measured rate of the color change indicates high sensitivity and first-order kinetics over a hydrogen peroxide concentration range of approximately 2 to 31 ppm. These new materials are a starting point for the preparation of more active sensor materials for hydrogen peroxide and organic peroxide vapor detection.

## 1. Introduction

Hydrogen peroxide vapor detection by using colorimetric sensing relies mostly on chemical binding or complexation-induced color changes [1,2,3,4]. This demonstrates a simple but very sensitive technique, which is used for chemical detection [5]. There are two-dimensional driving forces for this work. Firstly, this study aims to expand the fundamental science of colorimetric sensing for hydrogen peroxide vapor detection. Secondly, it aims to develop a method for H_2_O_2_ vapor detection, which remains challenging for recent sensing-based techniques. In addition, the development of a colorimetric sensing material based on complexation formation represents a simple, widely available, and cheap approach, which may lead to the manufacturing of portable and disposable devices [6].

Silicon-based TLC test strips, alumina-based test strips, as well as fiberglass blanket-type materials were used as a substrate instead of cellulose-based test strips. Silica test strips with acid were chosen, because acid destroys cellulose and alumina-based test strips, as shown in previous studies. Further studies using fiberglass blanket-type materials were conducted, which mimic the materials used in recent studies with lower sensitivity. Silica strips, made by Baker-flex, were used as the supporting material for all sample solutions and blanks. The 25 mm by 75 mm strips were cut into strips, and the silica was removed, leaving two 5 mm × 5 mm silica pads each. Each pad was separated by an empty space, because all the incoming vapors would not stay on the pad otherwise.

Hydrogen peroxide vapor detection has applications in industrial, bio-related monitoring and in the detection of peroxide explosives. One of the most widely used organic peroxide explosive is known as triacetone triperoxide (TATP), from which hydrogen peroxide vapor can be found as a compulsory compound [7,8,9]. TATP has no application for commercial or military purposes, because it has a tendency to sublime within a few days. Organic peroxide explosives are much more deadly compared to conventional high explosives. Furthermore, improvised organic explosive devices (IEDs) can be manufactured very easily and cheaply at home. However, conventional electronic detection systems are inactive when detecting these explosives through direct sensing [10]. Even fluorescence-based sensing materials fail to detect these peroxide-based explosives. For the detection of these deadly explosive materials, we need an advanced sensor material.

The mechanism of the advanced vapor deposition technique shows that the three-dimensional porosity of film materials allows for the diffusion of these vapor molecules throughout the materials’ matrix. Due to this diffusion of vapor materials throughout the host materials, a rapid vapor collection and accumulation is observed. A rapid response is always crucial in the detection of explosive chemicals, specifically for threatening and hazardous vapors [11,12,13,14]. In addition, a previous report shows that a fast sensor response was recorded in the time frame of seconds or even milliseconds [15]. Turbulent atmospheric flows can make this diffusion process extremely perturbed, causing large fluctuations in concentration with space and time [16].

The primary OSHA-approved method for measuring gaseous peroxide concentrations involves passing the atmosphere, under deliberation, through an acidified Ti(IV) oxysulfate solution [17]. The solution’s optical properties are then measured at a wavelength of about 410 nm to determine the peroxide’s concentration. For the purpose of quantifying gaseous peroxide, this method is still regarded as the ideal laboratory standard methodology. The use of hydrogen-peroxide-specific Dräger tubes, in which a color change over the course of a stationary phase is detected to show the peroxide concentration, is another less precise method [18]. Additional techniques for measuring and detecting peroxide include fluorometric [19], amperometric [20], potentiometric, chemiresistive, impedimetric, spectroscphosphorescence-based chemiluminescent, and photoluminescent techniques, which include fluorescence and phosphorescence-based sensors and electrochemical sensing [21,22]. Many of these techniques are difficult to automate, because in order to keep the instrument stable, they necessitate a pre-concentration of the atmosphere in the solution, some wet chemistry, or constant care from a technician.

Ionic liquids are salts that are typically liquid at or near room temperature. The fabrication and handling of ionic liquids, like Ti(IV) oxysulfate and ionic liquids, involve certain thermal conditions. The preparation of ionic liquids often requires heating under an inert atmosphere to prevent moisture or air contamination. Ionic liquids have been prepared at temperatures ranging from 100 °C to 300 °C. Ionic liquids often need to be stored at moderate temperatures (often below 100 °C) to maintain stability and avoid decomposition, as some can degrade or crystallize at elevated temperatures.

Higher temperatures are typically required to promote the reaction between titanium dioxide and sulfuric acid, allowing for the formation of Ti(IV) oxysulfate [23,24]. A controlled, high-temperature environment helps to break down the TiO_2_ structure and facilitates the incorporation of sulfur into the titanium dioxide matrix. A typical temperature range for this synthesis process is 100 °C to 250 °C. Depending on the desired phase of Ti(IV) oxysulfate, further thermal processing, such as calcination at temperatures between 300 °C and 600 °C, might be required to convert intermediate compounds into a stable Ti(IV) oxysulfate form. TiO_2_ has been heated with concentrated sulfuric acid in a controlled environment, usually at temperatures ranging from 100 °C to 250 °C.

Trifluoromethanesulfonic acid (TFMSA) is a strong organic acid, and its adsorption onto low-cost silicon thin-layer chromatography (TLC) plates can be influenced by thermal conditions: TFMSA can be adsorbed onto TLC plates at ambient temperatures. This adsorption process typically takes place under mild conditions, without significant heat being applied. The rate of adsorption can be affected by the temperature. Slight heating (up to 60 °C) can enhance adsorption by increasing the mobility of molecules, but excessive heat (above 100 °C) might result in the decomposition or evaporation of TFMSA. If desorption or recovery of TFMSA from the TLC plate is necessary, gentle heating (around 80 °C to 120 °C) can be used to facilitate the process.

H_2_O_2_ vapor detection is challenging for conventional sensing materials, but this issue has been resolved very carefully by a recently developed sensory material, which detects H_2_O_2_ vapors at the parts-per-million level. This sensory material is based on a titania-ionic liquid thin film. Ti(IV) oxysulfate, adsorbed on thin films, can be used for the detection of hydrogen peroxide vapor. Ti(IV) oxysulfate reacts and binds with H_2_O_2_ to form a Ti(IV)–peroxide complex, which causes the complex to change from being colorless to deep yellow or orange with an absorbance maximum peak of around 410 nm [25,26,27]. This color shift is due to complexation, which is highly selective for H_2_O_2_, because there is no color change when water; oxygen; typical organic reagents, for example, alcohols, acetone, and hexane; and chelating reagents are present.

## 2. Materials and Methods

### 2.1. Preparation of Titanyl-Ionic Liquid Solution with Acid

At the beginning of the synthesis, 1.69 g (approximately 1.69 mL) of H_2_O with 0.58 g (approximately 0.346 µL) of triflouromethanesulfonic acid was added in a test tube. Then, 0.5 g of solid titanium (IV) oxysulfate (TiOSO_4_·XH_2_O, purchased from Strem Chemicals and dried overnight in a vacuum desiccator) was added and immersed in a gently boiling water bath for 5 min to dissolve the solid substance in the test tube. Then, we waited for a clear solution to form in the test tube. After that, the solution was cooled at room temperature and filtered through a 0.20 microliter syringe filter paper to remove suspended particles. Then, 0.81 g (approximately 0.597 µL) of 1-ethyl-3-methylimidazolium hydrogen sulfate, known as so-called ionic liquid, was added and was mixed well inside the test tube. Then, this ionic liquid solution was stored in a vial after making it inert.

After preparing the sample solution, 20 µL of the sample solution was carefully placed into the silicon pad that contained silica as a substrate. Then, this amount was adjusted to 20 µL to fit the boxes on the pad, and a nice thin film was prepared. Then, the thin film was placed in the experimental setup, namely, in the exposure chamber of the experimental setup, and the sample was exposed to different concentrations of hydrogen peroxide vapor. The total concentration of hydrogen peroxide vapor was measured in the ppm range by measuring the absorbance of the colored solution into bubbler (E) of the experimental setup.

### 2.2. Preparation of Titanyl-Ionic Liquid Solution with Excess Acid

The same procedure was used for the preparation of a titanyl-ionic liquid solution with excess acid, except there was excess triflouromethanesulfonic acid, which was taken in the test tube during synthesis. A silicon-based TLC test strip, alumina-based test strips, as well as fiberglass blanket-type materials were used as a substrate.

### 2.3. Preparation of Titanium (IV) Oxysulfate and Peroxide Solution

#### 2.3.1. Titanium (IV) Oxysulfate Stock Solution

The hydrogen peroxide vapor concentration was measured through a so-called colorimetric procedure formed by OSHA. This method requires the preparation of a titanium (IV) oxysulfate stock solution in sulfuric acid. First, 5.5 g of dried TiOSO_4_∙XH_2_O (Strem Chemicals, Newburyport, MA, USA) was added to a beaker, and then, 20 g of ammonium sulfate (Spectrum, A.C.S. Reagent, La Mirada, CA, USA) was mixed in the beaker with a heated solution of 100 mL of concentrated sulfuric acid (Pharmco-Aaper, A.C.S. Reagent, Brookfield, CT, USA). The mixture was heated to dissolve all the chemicals. Then, the mixture was cooled to room temperature, and after cooling, 350 mL of nano-pure water was added in the resulting solution. A 0.40 µm filter paper was used to remove any suspended particles, and then, finally, more nano-pure water was added to further dilute the titanium(IV) oxysulfate solution to make the total volume of the stock solution 500 mL. A titanium reagent, commonly referred to as a collecting solution, is this stock solution diluted to 1:50.

#### 2.3.2. Hydrogen Peroxide Stock Solution

First, 2 mL of 30% H_2_O_2_ was added to a 500 mL volumetric flask, and then, distilled water was added to fill up the flask up to the mark. A total of 2 mL of this stock solution was diluted to 200 mL with distilled water. This is the required hydrogen peroxide stock solution. Aliquots of this stock solution were used as standards.

#### 2.3.3. Preparation of Starch Solution

A starch solution was prepared by adding 2 g of water-soluble starch and 0.01 g of HgI2 to 30 mL of boiling water. After that, a paste was formed. This paste was added to 1 L of boiling water. After cooling, the indicator solution was stored in a 1000 mL volumetric flask.

#### 2.3.4. Standardization of 30% Hydrogen Peroxide Solution

The following solutions are required for H_2_O_2_ standardization:

(1) 2 M H_2_SO_4_;
(2) 1 M KI;
(3) 1 M (NH_4_)_6_Mo_7_O_24_·4H_2_O;
(4) 0.001 M Na_2_S_2_O_3_;
(5) Starch solution.

After preparing all the reagents, the following solutions were transferred to a 125 mL Erlenmeyer flask: a total of 10 mL of stock H_2_O_2_ solutions, 20 mL of water, 10 mL, 2 M of H_2_SO_4_, 3–4 drops of 1 M, a (NH_4_)_6_Mo_7_O_24_·4H_2_O solution, 6 mL, 1 M of KI. Then, 0.001 M of a Na_2_S_2_O_3_ solution was added from a burette to the Erlenmeyer flask. This solution was titrated until a pale-yellow color formed. Then, 1 mL of the starch solution was added, which formed a blue color solution. After that, a few drops of 0.001 M of the Na_2_S_2_O_3_ solution were added until a colorless solution formed. The end point was recorded.

#### 2.3.5. Analysis of Samples and Standards

**Figure 1:**
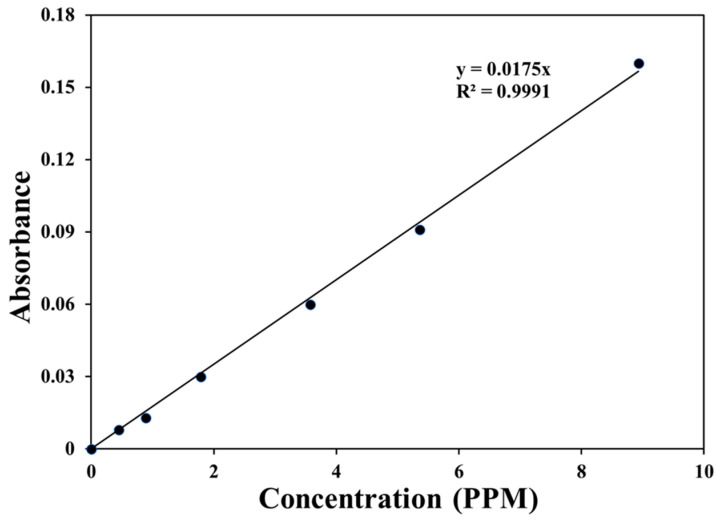
Calibration curve for the different concentrations of standard 30% H_2_O_2_ solution.

Standard H_2_O_2_ solutions were made by placing 5 mL of the titanium reagent in each of the 6 vials, and then, aliquots of the standard H_2_O_2_ solutions were added. The total volume was adjusted to 15 mL with nano-pure water. The absorbance of each sample solution, blank and standard, were determined at 410 nm with 100 cary UV–Vis spectrophotometer. An absorbance of 0.00 was measured for the blank reagent. After that, a standard calibration curve was plotted from the obtained absorbance values of the standard H_2_O_2_ solutions is shown in Figure 1. This standard curve should go through the origin.

#### 2.3.6. Film Testing Apparatus

**Figure 2:**
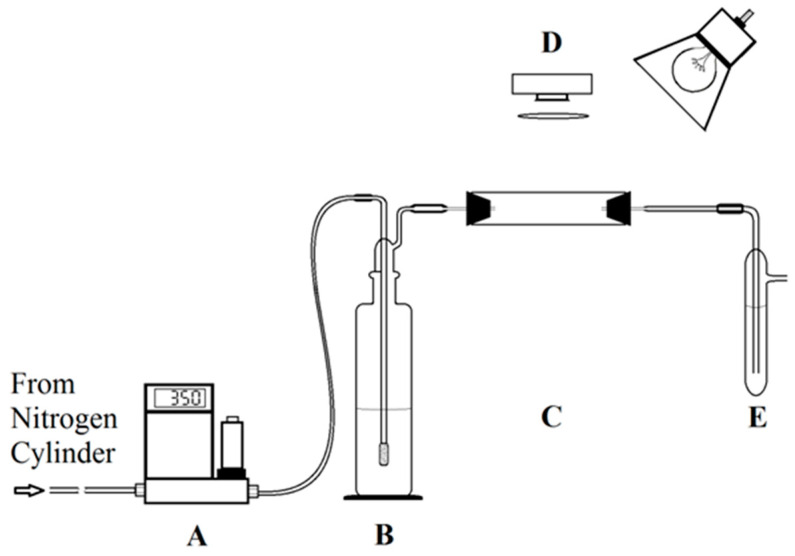
Schematic diagram of the apparatus that depicts the (**A**) flow controller, (**B**) bubbler to entrain the peroxide vapor, (**C**) exposure chamber, (**D**) detection system, and (**E**) a bubbler used to determine the total concentration of peroxide in the flow.

A schematic diagram of the experimental apparatus setup utilized to expose and track the thin films is shown in Figure 2. For the source of nitrogen as the carrier gas, a nitrogen cylinder was used, which was from (Airgas, Ultrahigh Purity, Radnor, PA, USA). The regulator back pressure was placed at 20 psi, and for all experiments, an Omega FMA 5500 mass flow controller (A) was used, where the flow was set to 350 standard cubic centimeters per minute (sccm). Then, a flow of nitrogen gas was run through a 250 mL bubbler (B) containing a peroxide solution. The gas was passed into the solution by using a sintered glass head, which spread the gas through the solution to ensure a steady absorption of the peroxide vapor. The hydrogen peroxide solution concentration in the bubbler defines the concentration of the gas phase, which is directly calculated as described below. After that, the peroxide-enriched gas was passed into a custom PTFE (Polytetrafluoroethylene) hollow cylinder cell containing the thin film, either a film on a silicon pad or a test strip (C). The PTFE cell contained a piece of white plastic paper attached to its outer end to generate a white background. A 20 W incandescent light was placed over the PTFE cell to deliver identical lighting for the slide along with images of the sample filmed with a Logitech Pro 9000 USB camera throughout introduction to the peroxide vapor (D). There was a Leiz Wetzlar BG 12 blue bandpass filter between the camera and the cell that separated the wavelengths of light between 300 and 500 nm.

The vapor was passed into a bubbler (E) when it left the PTFE cell and titanium(IV) oxysulfate solution, approximately 25 mL, in the bubbler, reacted with the peroxide vapor to produce a color change that was proportional to the total concentration of the peroxide vapor. There was another bubbler, parallel to the previous one, just to make sure that there was a complete reaction of the titanium(IV) oxysulfate solution with the dragged peroxide vapor. When the concentration of the peroxide vapor was 30% (*w*/*w*), no color change was observed in the parallel bubbler, and the exposure was for 30 min, which indicates that the reaction was completed without reaching the next bubbler.

A modified OSHA colorimetric procedure was used to determine the total peroxide vapor concentration by measuring the absorption values at a wavelength maximum λmax of 410 nm. Before measuring the absorption values by using this procedure, the UV–VIS spectrophotometer was calibrated, and all the measurements was conducted by using a cary 100 spectrophotometer. The vapor phase peroxide concentration (*v*/*v*) was measured based on the total concentration in the titanium(IV) oxysulfate solution at bubbler (E), and during all the experiments, a constant flow rate was maintained, which was 350 sccm, and also, the exposure time of the vapor to the thin films was constant. Several concentrated solutions of sample peroxide were used, which were prepared from the 30% (*w*/*w*) peroxide solution, and during our experiments, each time, this procedure was used to measure the peroxide vapor concentration at (*v*/*v*). The OSHA VI-6 procedure was used as a reference for the quantification of the peroxide vapor of the sample solution.

The total N_2_ gas volume that passed through the experimental setup during the experiments was measured at 21 L for a 1 h period of exposure. One of the characteristics of N_2_ gas is that it forms bubbles through a hydrogen peroxide solution. Therefore, if we add additional water vapor, this could enhance the total gas volume slightly. No additional water vapor was added during our experiments, so a marginal underestimation of the vapor concentration could occur. When the bubbler was used for an extended period of time, approximately 18 h, there was a loss of mass of water, which was around 0.5 g/h. If we convert this mass into volume, which will be around 0.6 L of water vapor within 21 L of N_2_ gas, its contribution towards lowering the error towards the ppm value was approximately 3%.

As was mentioned before, there were several different concentrations of the hydrogen peroxide solution for each experiment that gave different peroxide vapor concentrations in the bubbler. The exact concentration was measured by taking several attempts with the same sample solution. For every exposure of peroxide vapor, an image was formed after each 30 s and was recorded and saved by using a python coding setup. The whole framework took 1 h for each peroxide vapor exposure. After generating all the images, the images were analyzed using the ImageJ software (1.53k, 2021) in order to plot an intensity vs. time plot. The intensity was obtained by selecting several center-section areas of the image, and the total intensity plots were generated by this process. The wavelength range was 300–500 nm during the experiment, and a BG 12 blue bandpass filter was used to obtain this wavelength range. In addition, a monochromator and tungsten lamp were used to produce a monochromatic broadband light, which also showed less sensitivity for the red light in the UV region, or blue region. Therefore, saturation occured at the blue region of the reflected light, and the red region was used to measure the reflected light.

## 3. Data Acquisition and Analysis

**Figure 3:**
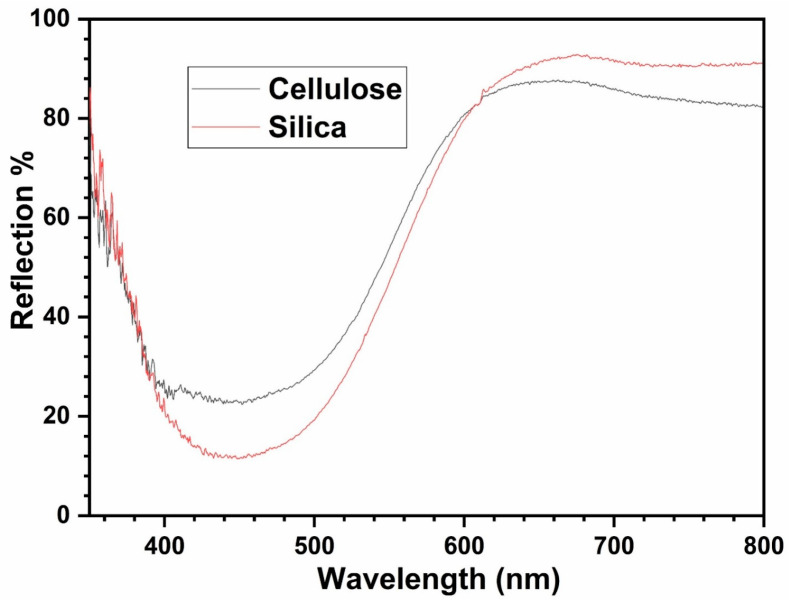
UV–vis reflection spectra for silica and cellulose test strips.

Different titanyl-ionic liquid solutions with acid were exposed to liquid H_2_O_2_ and then were deposited on various substrates (silicon- and cellulose-based test strips), and their reflected spectra were acquired using a spectrometer as shown in Figure 3. The data were collected using a spectrometer with sampling points from 350 to 800 nm and a sampling contact probe consisting of a halogen lamp and a collecting fiber as the input.

**Figure 4:**
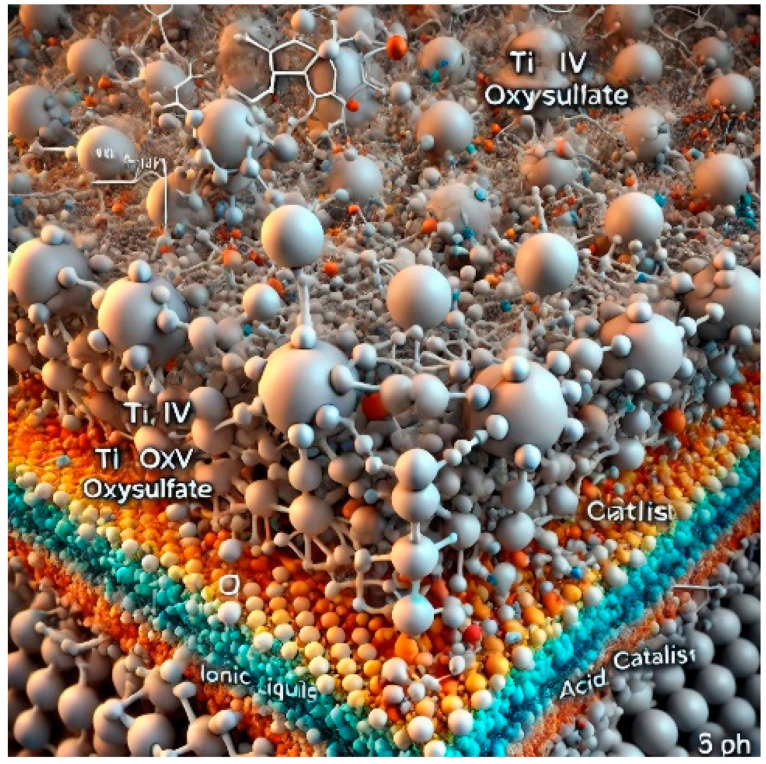
Visualization showing the interaction of Ti(IV) oxysulfate, ionic liquids, and an acid catalyst at the microstructural level.

For each substrate, various measurements were performed, having different concentrations of peroxide per unit surface. Thus, a large dataset was collected, comprising of a range of intensities and different relations between absorption and reflection peaks. Another source of spectral diversity is the pH of the measured samples, which affects their reflecting properties. It is worth noticing that due to these pH effects, there was a red shift in the reflected spectra is shown in Figure 4. Therefore, by examining the wavelength of the reflected light, information about complex formation and color changes can be obtained.

A scientific visualization at the microstructural level illustrates the interaction among Ti(IV) oxysulfate, ionic liquids, and an acid catalyst, revealing a hybrid structure characterized by well-distributed components. The visualization highlights spherical particles of Ti(IV) oxysulfate as the primary structural units, surrounded by an amorphous layer, attributed to the ionic liquid is shown in Figure 4. This amorphous matrix provides a uniform medium that stabilizes the Ti(IV) oxysulfate particles and prevents agglomeration, as no evidence of particle clustering is observed. The dispersed nature of the acid catalyst molecules within this matrix further emphasizes the homogeneous distribution of all components, promoting effective interfacial interactions.

The interplay between these components is central to the formation of the hybrid structure. The ionic liquid appears to act as a mediator, encapsulating the Ti(IV) oxysulfate particles and providing a flexible matrix that accommodates the acid catalyst. This configuration suggests potential synergy between the crystalline Ti(IV) oxysulfate particles and the surrounding amorphous phases. This visualization thus provides critical insights into the structural organization and interactions that highlight the material’s hybrid nature and performance.

**Figure 5:**
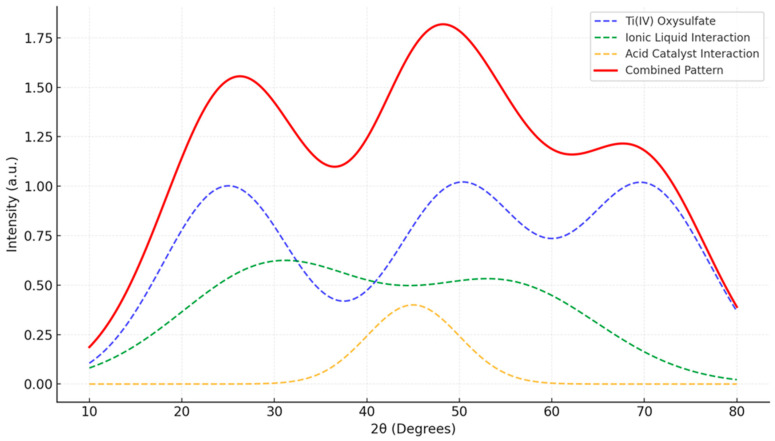
XRD pattern of Ti(IV) oxysulfate with ionic liquid and an acid catalyst.

An X-ray diffraction (XRD) analysis was conducted to study the structural interactions between Ti(IV) oxysulfate, ionic liquids, and an acid catalyst, revealing a combination of crystalline and amorphous features that highlight the hybrid nature of the material see Figure 5. The sharp and well-defined peaks associated with Ti(IV) oxysulfate confirm its crystalline structure, indicating the retention of ordered domains within the material. In contrast, the ionic liquid introduces a degree of structural disorder, as evidenced by the broadening of certain peaks. This broadening suggests partial amorphization or a disruption of the crystalline lattice, likely due to the incorporation of ionic liquid components into the material.

The acid catalyst further contributes to the structural complexity, as indicated by an additional broad peak, which is characteristic of an amorphous phase or chemical modifications to the material’s structure. These features suggest that the acid catalyst promotes changes in the local arrangement of atoms, potentially through the formation of hydrogen bonds or other interactions. The combined XRD pattern, with its mixture of sharp crystalline peaks and broadened amorphous features, reflects the hybrid nature of the material. This structural interplay between crystalline and amorphous domains likely enhances the functional properties of the system, making it suitable for applications requiring a combination of ordered and disordered structural characteristics.

**Figure 6:**
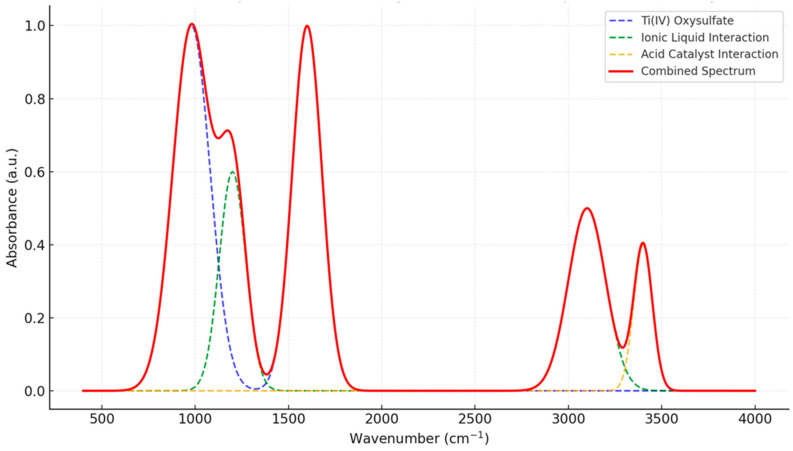
FTIR spectrum of Ti(IV) oxysulfate with ionic liquid and an acid catalyst.

Fourier-transform infrared (FTIR) spectroscopy was utilized to investigate the interactions among Ti(IV) oxysulfate, ionic liquids, and an acid catalyst, revealing distinct absorption bands indicative of chemical and physical interactions. The Ti(IV) oxysulfate contribution is evident from strong absorption peaks near 980 cm^−^^1^, corresponding to Ti–O stretching vibrations, and at ~1600 cm^−^^1^, attributed to O–H bending modes. These features highlight the characteristic structural motifs of Ti(IV) oxysulfate see Figure 6. The ionic liquid introduces new vibrational bands, including peaks around 1200 cm^−^^1^, which can be assigned to C–N or C–O stretching modes, and a band near 3100 cm^−^^1^, corresponding to C–H stretching vibrations, likely arising from the alkyl chains or imidazolium cations of the ionic liquid. The acid catalyst contributes a broad absorption band centered at ~3400 cm^−^^1^, associated with O–H stretching vibrations, indicative of hydrogen-bonding interactions or hydroxyl groups.

The spectrum reflects the overlapping and emergent features arising from the interaction of these components. The introduction of new peaks and broadening of specific regions suggest potential bonding or coordination between Ti(IV) centers and ionic liquid or acid functional groups. Additionally, the hydrogen bonding inferred from the broad absorption near 3400 cm^−^^1^ implies significant structural reorganization within the system. Collectively, these observations provide strong evidence for chemical and physical interactions among Ti(IV) oxysulfate, ionic liquid, and the acid catalyst, which play a crucial role in determining the functionality and reactivity of the system.

**Figure 7:**
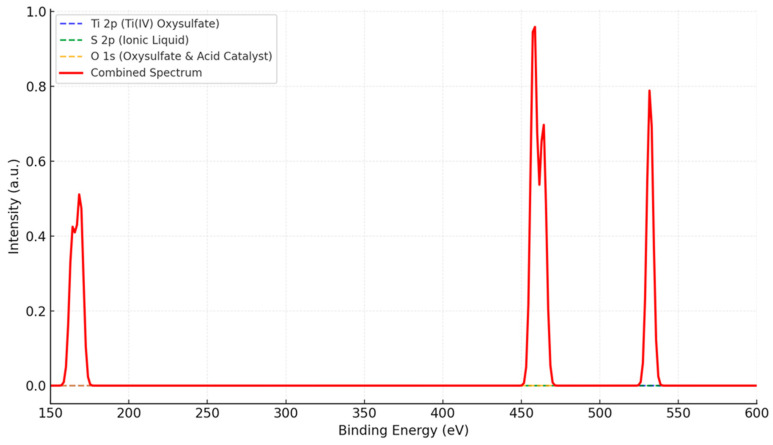
XPS spectrum of Ti(IV) oxysulfate with ionic liquid and an acid catalyst.

The X-ray photoelectron spectroscopy (XPS) analysis of the interaction between Ti(IV) oxysulfate, ionic liquid, and an acid catalyst reveals distinct core-level peaks, offering insights into the chemical states and interactions of the components. The Ti 2p region displays characteristic peaks at approximately 458 eV (Ti 2p_3_/_2_) and 464 eV (Ti 2p_1_/_2_), indicative of the presence of Ti(IV) species see Figure 7. In the S 2p region, peaks in the range of ~164–169 eV suggest contributions from sulfur species associated with the ionic liquid and/or the oxysulfate. A prominent O 1s peak at ~532 eV is observed, reflecting oxygen contributions from the oxysulfate and from the acid catalyst. The combined spectral data highlights the presence of titanium, sulfur, and oxygen in their expected chemical states and suggest interactions and potential bonding between these components. The observed shifts and intensities in the spectra provide direct evidence of the chemical environment’s influence on stability and reactivity.

**Figure 8:**
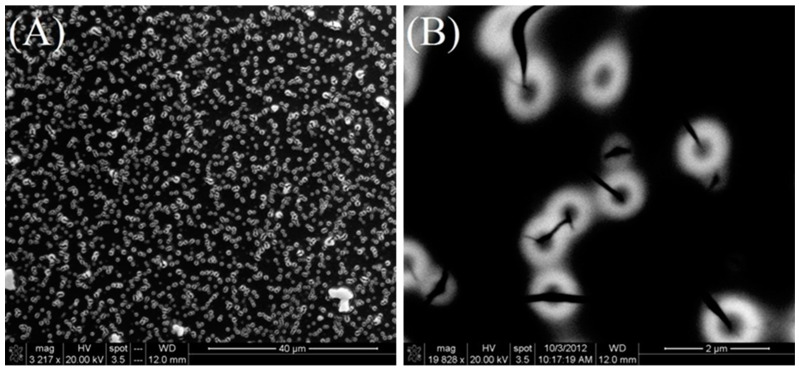
Scanning electron micrograph of two-week-old titania film at magnifications of (**A**) 3000× and (**B**) 20,000×.

To further verify the morphology and particle size distribution, scanning electron microscopy (SEM) was used as a characterization technique. The SEM analysis was conducted on a film prepared from a solution aged for two weeks. High-resolution SEM imaging revealed a dominant population of titanium particles exhibiting a uniform spherical morphology with an average diameter of approximately 1 μm is shown in Figure 8. The uniformity in size suggests consistent nucleation and growth processes during the formation of the particles, which could be attributed to controlled solution conditions and aging times. This observation corroborates the effectiveness of the synthesis method in producing monodisperse titanium particles suitable for applications requiring precise size control.

(1) Microstructural Consistency (SEM and TEM Insights)

The visualization and SEM features indicate well-distributed Ti(IV) oxysulfate particles embedded in the ionic liquid matrix with no evident agglomeration. The structure appears uniform with interactions at the nano scale, supporting compatibility between the components.

(2) Crystallinity and Phase Consistency (XRD Analysis)

The XRD pattern shows characteristic Ti(IV) oxysulfate peaks retained with some broadening and minor shifts, indicating partial amorphization due to interactions with the ionic liquid and acid catalyst. The phase modification was controlled and consistent with expected chemical and physical interactions.

(3) Functional Group Interactions (FTIR Analysis)

The FTIR spectrum shows retention of Ti–O and O–H functional groups from the oxysulfate and acid. New peaks occur at ~1200 cm^−^^1^ and ~3100 cm^−^^1^, indicating interactions with the ionic liquid and potential hydrogen bonding. The functional groups and chemical interactions align with expectations, showing stable and interactive behavior.

(4) Chemical State and Stability (XPS Analysis)

The XPS spectrum reveals Ti 2p peaks (~458 and 464 eV), confirming the presence of Ti(IV). The S 2p peaks (~164–169 eV) and O 1s peak (~532 eV) reflect contributions from the ionic liquid and oxysulfate. No unexpected shifts or additional peaks that would indicate decomposition or side reactions occurred. The system’s chemical states remained stable and consistent.

This indicates that the system was well-integrated and retained the desired microstructural and chemical properties.

## 4. Results and Discussion

### 4.1. Reaction Kinetics with Different Substrates

Hydrogen peroxide vapor was exposed to the thin films, and changes in color were observed, wherein intensity was a function of peroxide concentrations as well as the exposure period. As discussed in the experimental chapter, saturation occurs in the blue region as a consequence of images that were initially saturated at that region. During the reaction of the exposed vapor and thin films, UV spectra of exposed thin films were formed within a 300 to 500 nm wavelength region, and colored images resulted. There was no color change when a controlled thin film was exposed to hydrogen peroxide vapors without adding any titania.

**Figure 9:**
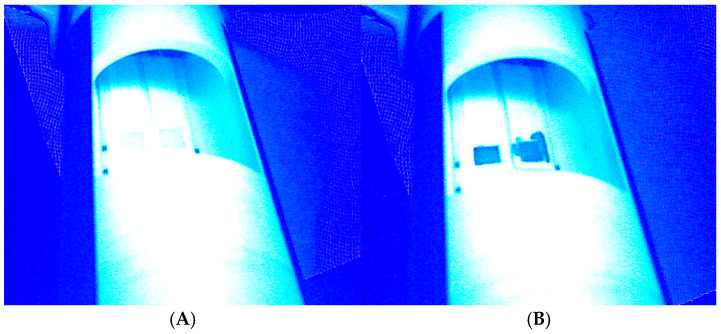
Reflected images of thin film (**A**) before and (**B**) after peroxide exposure. The thin film was exposed to a hydrogen peroxide vapor concentration of 30.9 ppm for 1 h.

A 1/8-m monochromator and tungsten lamp were used to produce monochromatic broadband light and gave rational absorbance spectra, which were obtained by using a web camera that had red dye. Hence, a lower sensitive but more dynamic range was observed for the red region. By using the ImageJ software and dynamic red region, reflected images were obtained for the thin films, as shown in Figure 9.

**Figure 10:**
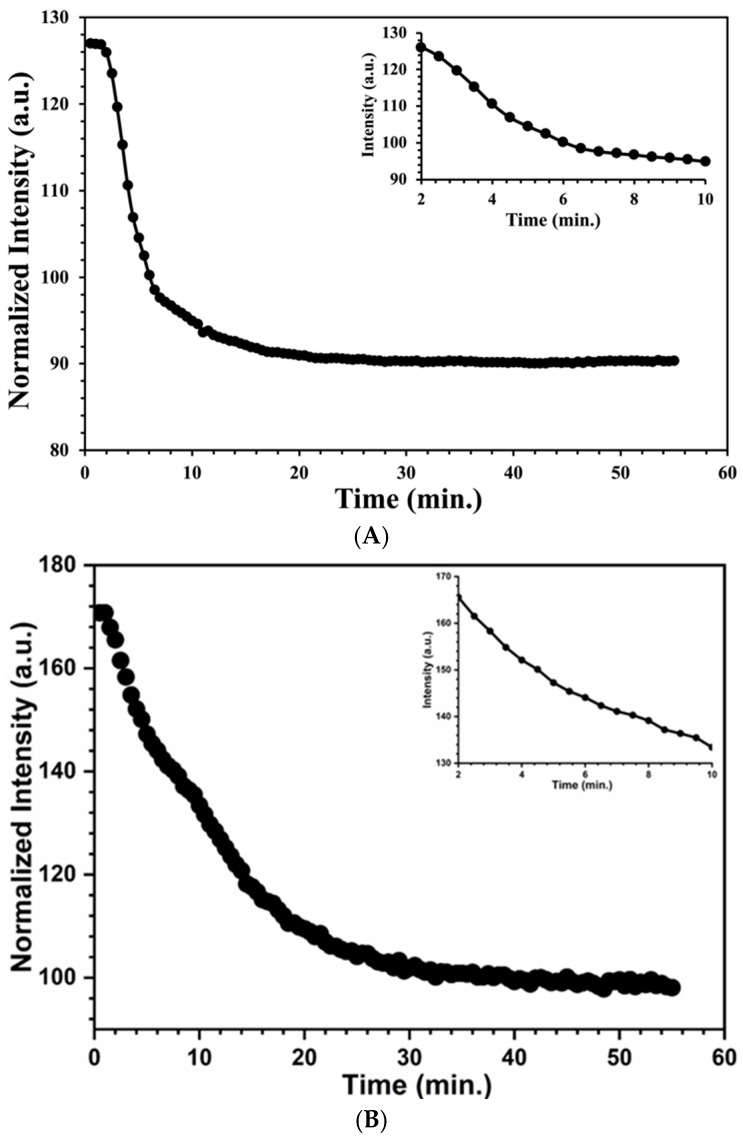
(**A**) Intensity versus exposure time for a cellulose test strip; the insert shows a blowup of the region of rapid decrease in intensity between 2 and 10 min; (**B**) the intensity versus exposure time for a silica test strip—with acid; the insert shows a blowup of the region of rapid decrease in intensity between 2 and 10 min.

**Figure 11:**
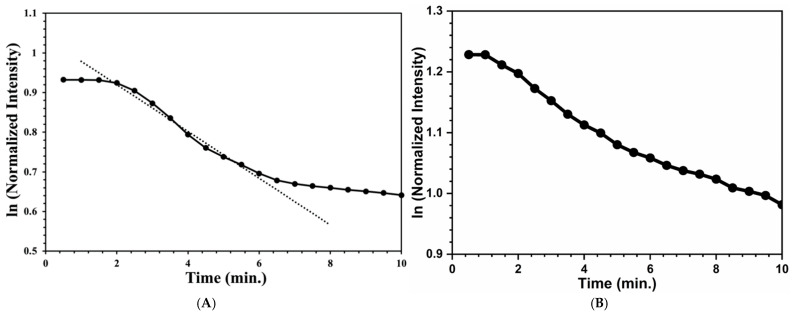
(**A**) The first-order behavior of the first 10 min of exposure. The cellulose test strip was exposed to a peroxide concentration of 28 ppm for 1 h and (**B**) the first-order behavior of the first 10 min of exposure. The silica test strip was exposed to a peroxide concentration of 30.9 ppm for 1 h.

As discussed in the experimental section, after forming all the images, the images were analyzed using the ImageJ software in order to plot an intensity vs. time plot. There was a decrease in reflected intensity as an increase in time, as shown in Figure 10 for both test strips. Upon exposure of the peroxide vapor, the intensity usually decreases exponentially with time. Another plot was obtained for the natural logarithm of the ln (intensity) with exposure time, Figure 11, which was a linear plot, and which was exponentially time dependent. The included figure here is an experimental run with a 28 and 30.9 ppm hydrogen peroxide vapor exposure on cellulose and silica-based test strips. In addition, several different experimental runs with different hydrogen peroxide concentrations were accomplished.

A first-order kinetic behavior was chosen over other orders, as it is the simplest form that describes the experimental data and allows for a comparison with previous data. By using the rate constant equation and from the plot, a negative slope was obtained from Figure 11. In addition, the linear regression of the plots was measured. When saturation occurred, the reflected intensity also stopped, as there were no active titania materials that remained to interact with the peroxide vapor. It was observed that when there was a higher loading of the titania materials, this produced a smaller intensity, because more light was absorbed rather than reflected. For each experiment, the saturation intensities were monitored by measuring the average of the final five minutes of vapor exposure, and it was found that the difference was only less than 1% from the calculated previous run.

**Figure 12:**
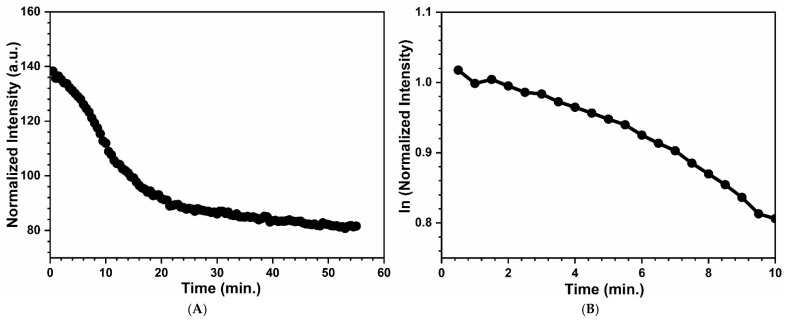
(**A**) Intensity versus exposure time for a silica test strip—without acid and (**B**) the first-order behavior of the first 10 min of exposure. The silica test strip was exposed to a peroxide concentration of 29 ppm for 1 h.

On the other hand, the decrease in reflected intensity with an increasing time is shown in Figure 12A for the silica test strip without acid. Upon exposure of the peroxide vapor, the intensity usually decreases exponentially with time. However, for the without-acid plot, there was an unexpected bump in the intensity vs. time plot that could be due to the side reaction of sulfur with the peroxide, or it could be the formation of different peroxide and Ti complexes, which was not further analyzed. The natural logarithm plots were obtained for the ln (intensity) vs. exposure time, shown in Figure 12B, which resulted in linear plots, indicating a first-order reaction and an exponential time dependence. The included figures are an experimental run with 29 ppm of hydrogen peroxide exposure.

In addition, several different experimental runs with different hydrogen peroxide concentrations were accomplished. Loading of the ionic liquid sample solution on the test strips is important for the magnitude of the final absorption and reflection values, because if there is higher titania loading, then the intensity change will be smaller as a result of the greater amount of light absorption. The silica substrate is also a very important material as a support, because this silica substrate does not react with the acid.

**Figure 13:**
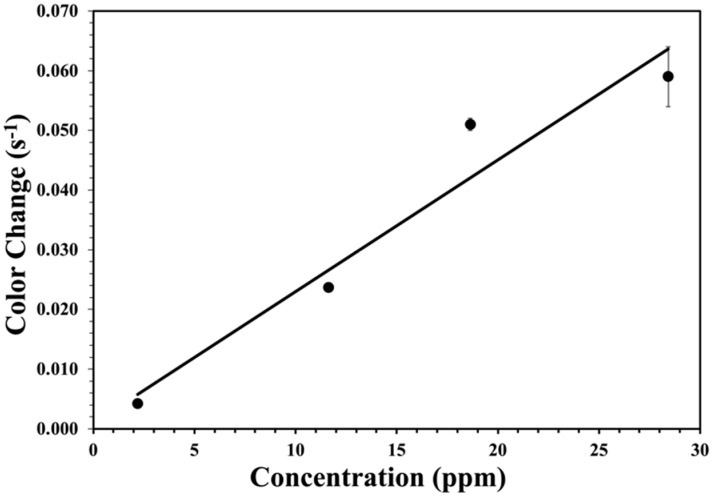
Phenomenological first-order rate constants obtained from the test strip as a function of the peroxide concentrations. Error bars were determined from thin film homogeneity, as discussed in the text.

The phenomenological first-order rate constants versus measured peroxide vapor concentrations in ppm for the cellulose test strip are shown in Figure 13. Inhomogeneity was the main reason for the experimental error. The test strip was divided into four same-sized sections to measure the error bars and rate constants in Figure 13, as well as to find the standard deviation for the rate constant in each section. There was a linear relationship between the rate constants and measured peroxide vapor concentrations. Therefore, on the surface, having a constant concentration of the titanium(IV) species could change the color linearly, proportional to the flux of the peroxide vapor. The linear regression value for the phenomenological first-order rate constants versus measured hydrogen peroxide vapor concentrations was obtained for the cellulose test strip with an R^2^ of 0.94.

The resulting color change for the cellulose test strip without acid is 2.2 ± 0.3 × 10^−3^, from Figure 13. The resulting color change for the silica test strip with acid is 1.3 ± 0.04 × 10^−3^ color change per ppm per second. In fact, as can be seen from both figures, the slope of the regression line for the cellulose test strip is 2.2 × 10^−3^, and the slope for the silica test strip is 1.3 × 10^−3^, but is this difference significant?

To compare the similarity of the slopes, a *p*-value approach was used. A probability value of less than 0.05 indicates that the two slopes are significantly different from each other. The *t*-value is calculated using the following equation.

$$t=\frac{b_{1}-b_{2}}{\sqrt{s_{b_{1}}^{2}+s_{b_{2}}^{2}}}$$

*b*_1_ and *b*_2_: the two means you are comparing, one from each dataset.

$\sqrt{s_{b_{1}}^{2}+s_{b_{2}}^{2}}$: the combined standard error of the two samples (calculated using pooled or unpooled standard errors)

From the calculation, *t*-value = 2.97

Degrees of freedom = 4

Probability = 0.041

Thus, the probability is 0.041. As this value is less than 0.05, the two slopes are significantly different from each other.

**Figure 14:**
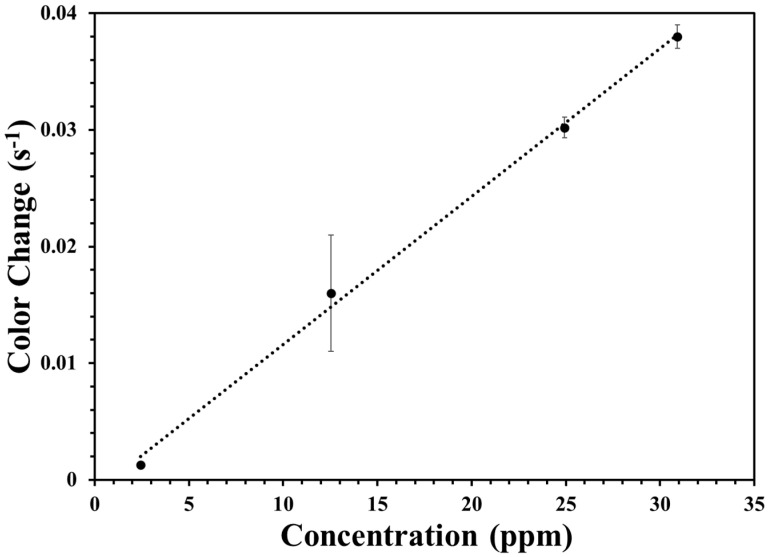
Phenomenological first-order rate constants obtained from the silica test strip with acid as a function of the peroxide concentrations. Error bars were determined from thin film homogeneity, as discussed in the text.

The phenomenological first-order rate constants versus measured peroxide vapor concentrations in ppm for the silica test strip with acid is shown in Figure 14. The linear regression value for the phenomenological first-order rate constants versus measured hydrogen peroxide vapor concentrations for with acid plots was obtained for the silica test strips with an R^2^ of 0.99. The value of the slope from Figure 14 is 1.3 ± 0.04 × 10^−3^, which is the color change per ppm per second. The results obtained suggest that the silica-based test strip shows approximately identical sensitivity with the cellulose-based test strip. This means that both the test strips are superior materials with almost equal sensitivity and reproducibility. The surface area and porosity of the cellulose test strip is comparatively higher than the silica test strip, and the porous nature of the test strip is not likely the cause of the different rates. The volume of the titania sample also plays a crucial role for the different rates of these thin films. Usually, when there was a higher load of titania materials, more H_2_O_2_ vapor could be captured and complexed at a certain time interval, which leads to an increased sensing efficiency. However, after the optimum loading, a further loading of titania could inhibit the porosity, resulting in a decrease in the vapor penetration. Both types of test strips were white-colored; therefore, no added background materials were needed to reflect the light. Furthermore, based on the experimental setup, there could be some differences in rates between the test strips. The control thin films with no added titania on the test strips showed no reactivity towards the peroxide vapor, which also can be referred to as the background.

The kinetic parameters for both types of test strips on the rate of color change are presented in Table 1, assuming first-order kinetics. From the table, it can be seen that the linear regression value was 0.94 and 0.99 for the test strip without and with acid, respectively. The slope of the first-order rate constant was 0.059 and 0.038 for the cellulose and silica test strip. The concentration of the hydrogen peroxide vapor was 28.4 and 30.9 in (*v*/*v*).

### 4.2. Peroxide Sensitivity and Reaction Mechanism

The first-order rate constants versus measured peroxide vapor concentration plots, Figure 13 and Figure 14, can be explained by a reaction mechanism, which relies on the flux of hydrogen peroxide vapor on the surface. The first-order rate constants obtained at different peroxide vapor concentrations heavily rely only on the flux of the H_2_O_2_ vapor to the surface. Therefore, the hypothesis is further supported by the constant concentration of the titanium (IV) species on the surface that could change color, which is linearly proportional to the flux of the hydrogen peroxide vapor or partial pressure. The rate of formation of the colored species is relatively faster at the surface than the arrival rate. It is the concentration of existing reactive sites, apparently the encapsulated titanium (IV) species within both types of test strips, which is the main reason behind the final coloration that is entirely used at later time points. The fast reaction rate at the surface suggests that, at these various hydrogen peroxide vapor concentrations, the obtained rate should not depend on the number of reactive sites within the test strips. However, the highest capacity of these test strips is restricted by the number of reactive sites supplied by the compressed titanium(IV) species at the surface; hence, when the loading is higher, a greater definitive color change occurs on the presence of the hydrogen peroxide vapor. Furthermore, this model is consistent with the freedom of the phenomenological rate constant with the titania loading in Figure 13 and Figure 14.

## 5. Conclusions

In conclusion, the development of sensors capable of effectively detecting hydrogen peroxide vapor is significantly less advanced compared to solution-based detection techniques. The primary challenge lies in collecting and detecting hydrogen peroxide in its vapor phase, especially in an open atmospheric condition, where the substance rapidly disperses. Existing sensing materials lack the capability for the fast adsorption and accumulation of hydrogen peroxide vapor immediately upon exposure. While some materials demonstrate a degree of sensitivity, their performance falls short in terms of rapid collection, efficiency, and reliability, highlighting the critical need for further advancements in chemical-based sensors.

The test strip materials used in the experiments exhibited a promising level of reactivity toward hydrogen peroxide vapor, with visible color changes—ranging from intense yellow to orange—when exposed under different acidic conditions. These changes indicate potential for semi-quantitative detection. However, to achieve greater accuracy and sensitivity, integrating advanced optical or electronic detection techniques could significantly enhance the ability to measure the reflected intensity of these color changes. Such improvements could lead to the development of highly sensitive and practical hydrogen peroxide vapor detectors.

Moreover, recent research efforts are expanding to investigate the response of these thin-film materials to organic peroxides. This line of inquiry not only broadens the scope of applications for the developed sensors but also has the potential to advance the field of peroxide vapor detection as a whole. These ongoing developments, if successful, could pave the way for more robust, efficient, and versatile detection systems suited to a variety of environmental and industrial contexts.

## Figures and Tables

**Table 1:** Kinetic parameters for the rate of color change on thin films.

First-Order Kinetic Model for Thin Film with Acid			Overall Order	First Order Kinetic Model for Thin Film Without Acid		
C (ppm)	k (S^−1^)	R^2^		C (ppm)	k (S^−1^)	R^2^
30.9	0.038 ± 0.001	0.99		28.4	0.059 ± 0.005	0.98
24.9	0.0302 ± 0.0009	0.99	1.0	18.6	0.051 ± 0.001	0.99
12.5	0.016 ± 0.005	0.96		11.6	0.0237 ± 0.0005	0.99
2.44	0.0013 ± 0.0001	0.92		2.2	0.0042 ± 0.0003	0.98

## Data Availability

The data presented in this study are available on request from the corresponding author.
